# Resting-State Functional Correlates of Social Cognition in Multiple Sclerosis: An Explorative Study

**DOI:** 10.3389/fnbeh.2019.00276

**Published:** 2020-02-06

**Authors:** Alvino Bisecco, Manuela Altieri, Gabriella Santangelo, Federica Di Nardo, Renato Docimo, Giuseppina Caiazzo, Rocco Capuano, Simona Pappacena, Alessandro d’Ambrosio, Simona Bonavita, Francesca Trojsi, Mario Cirillo, Fabrizio Esposito, Gioacchino Tedeschi, Antonio Gallo

**Affiliations:** ^1^Department of Advanced Medical and Surgical Sciences, University of Campania Luigi Vanvitelli, Napoli, Italy; ^2^Department of Psychology, University of Campania Luigi Vanvitelli, Caserta, Italy; ^3^Department of Medicine and Surgery, University of Salerno, Baronissi, Italy

**Keywords:** multiple sclerosis, social cognition, cognition, resting state functional connectivity, MRI

## Abstract

Social cognition includes mental operations essential for functional social interactions, and several studies revealed an impairment of social cognition abilities in patients with Multiple Sclerosis (MS). These deficits have been related to global and focal gray matter atrophy as well as microstructural white matter damage. Although some studies reveal a correlation between social cognition and task-based functional magnetic resonance imaging (MRI), no studies to date have explored the association between brain resting-state functional connectivity (RS-FC) abnormalities and several measures of social cognition in MS. The aim of this explorative study was to assess the contribution of RS-FC abnormalities of major brain networks to social cognition in MS patients. Clinical, neuropsychological, and MRI data were collected from 41 non-depressed and cognitively preserved relapsing-remitting MS patients (mean disease duration = 8.8 ± 8.2 years; median Expanded Disability Status Scale = 1.5, range 0–6.5) and 25 matched healthy controls (HCs). The ToM Pictures Sequencing Task (TMPS) and the Reading the Mind in the Eyes Task were employed to evaluate social cognition. All participants underwent a structural MRI and RS functional MRI 3T protocol. Regional gray matter atrophy was measured, and FCs of the default mode (DMN), right and left fronto-parietal, executive (EN), salience, cerebellar, and limbic (LN) networks were evaluated by independent component analysis (ICA). Differences on TMPS were found between MS patients and HC (MS < HC). In the MS group, associations were found between right middle temporal gyrus FC (in the DMN) and reciprocity subscale of TMPS, posterior cingulate cortex (PCC) FC (in the DMN) and first-order false-belief subscale of TMPS, cingulate gyrus FC (in the EN) and TMPS as well as reciprocity subscale of TMPS, and right superior temporal gyrus (in the LN) and reciprocity subscale of TMPS. All detected RS-FC changes did not co-localize with regional gray matter atrophy. The results suggest an association between social cognition and RS-FC changes of DMN, EN, and LN in MS. Future studies should further explore the possible adaptive or maladaptive mechanisms of these FC abnormalities in MS.

## Introduction

Social cognition includes mental operations essential for functional social interactions (Beer and Ochsner, 2006). A key aspect of social cognition is social understanding (Arioli et al., 2018), the cognitive ability to decode and attribute mental states such as goals or intentions, knowledge, belief, thoughts and emotions to oneself and others (Premack and Woodruff, 1978).

As regards the neural correlates of social cognition, a meta-analysis on task-based functional magnetic resonance imaging (fMRI) studies revealed a core network, including the medial prefrontal cortex and bilateral temporo-parietal junction, which are consistently activated independently from the type of the instrument employed to assess social cognition (Schurz et al., 2014). However, different cortical areas were found activated on the basis of specific tasks. For example, the above-mentioned meta-analytic study (Schurz et al., 2014) revealed that the dorsal/posterior parts of the temporo-parietal junction showed an increased connectivity during tasks that required processing of false-belief, whereas the ventral/anterior parts of the temporo-parietal junction were engaged for tasks that depicted rational actions or behaviors.

Social cognition deficits occur in several neurological diseases such as amyotrophic lateral sclerosis (Trojsi et al., 2017), Parkinson’s disease (PD; Bora et al., 2015), behavioral-variant frontotemporal dementia (Henry et al., 2014) and multiple sclerosis (MS; Henry et al., 2009, 2011; Banati et al., 2010; Pöttgen et al., 2013; Bora et al., 2016; Cotter et al., 2016). In MS, it is unclear whether (cognitive and affective) aspects of social cognition are both impaired: some studies found that the two subcomponents are equally impaired (Raimo et al., 2017), while others revealed deficits only on a single subcomponent, either cognitive (Roca et al., 2013) or affective (Cotter et al., 2016).

The neural substrates of deficit of social cognition in MS have been explored by means of MRI in several studies, revealing an association between social cognition and different measures of brain damage (Mike et al., 2013; Batista et al., 2017a,b; Chalah et al., 2017). In more detail, a worse performance on tests assessing social cognition has been related to reduced total and regional gray matter (GM) volumes, especially in the cingulate, orbitofrontal, and cerebellar cortex, as well as in the insula and the amygdala (Batista et al., 2017a; Chalah et al., 2017; Ciampi et al., 2018). As for the relationship between social cognition and white matter (WM) damage, a lower performance in tasks assessing social cognition was associated with macrostructural (T2 lesion volume; Mike et al., 2013; Batista et al., 2017b; Chalah et al., 2017) as well as microstructural (normal-appearing WM) damage, especially within tracts of limbic pathways and callosal interhemispheric fibers (Batista et al., 2017b), which are involved in social and communicative abilities or emotional processing (Paul et al., 2007; Von Der Heide et al., 2013; Downey et al., 2015). Few studies have used task-based fMRI to verify whether impairment on social cognition abilities was associated with a decreased or increased activation of specific cerebral area, and found that impairment of ability to recognize emotional facial expression was associated with decreased activation of the insular and ventrolateral prefrontal cortex (Jehna et al., 2011) or with an increased activation of the anterior and posterior cingulate cortex (PCC), praecuneus and occipital fusiform gyri (Krause et al., 2009). However, these studies have the limitation of investigating only specific aspects related to social cognition, such as facial emotion detection.

To date, no resting-state (RS) fMRI studies have explored cognitive and affective ToM in MS. RS fMRI represents a unique method to investigate brain networks with minimal bias, and it has been employed in several studies in MS (Gallo et al., 2012; Filippi and Rocca, 2013; Sbardella et al., 2015; Bisecco et al., 2018; Rocca et al., 2018). In particular, using resting-state functional connectivity (RS-FC) eliminates the nuisance effect of performance variability present during task-based fMRI studies. Therefore, the aim of our explorative study is to fill the above-mentioned knowledge gap by assessing the contribution of RS functional connectivity (RS-FC) abnormalities to social cognition in patients with MS. Since some studies report an association between social cognition and cognitive abilities, both in healthy (Apperly et al., 2009; Wade et al., 2018) and in MS patients (Raimo et al., 2017), we explored RS networks mainly associated with high-level cognitive and/or social abilities: (1) the default mode network (DMN), which actively supports several aspects of cognition, like working memory, memory retrieval, or divergent thinking (Spreng, 2012; Murphy et al., 2018); (2) the right and left fronto-parietal network (FPN), involved in cognitive control and in allocating attentional resources (Marek and Dosenbach, 2018); (3) the executive network (EN), engaged in high-level cognitive functions such as goal-directed behavior, working memory, and cognitive control (Menon, 2011); (4) the salience network (SLN), involved in the detection of relevant stimuli in the environment and in the coordination of behavioral responses (Chand and Dhamala, 2016); (5) the limbic network (LN), as amygdala lesions or atrophy was found to be associated with impairment of social understanding (Shaw et al., 2004; Batista et al., 2017a); and (6) the cerebellar network (CN), which is implicated in social cognition and in higher abstraction mentalizing (Van Overwalle et al., 2014).

## Materials and Methods

### Participants

Forty-one relapsing-remitting MS patients were consecutively recruited at the MS center of the Division of Neurology of the University of Campania “Luigi Vanvitelli,” in Naples (Italy); 25 Healthy controls (HCs) were recruited from a large HC database created in our Institution. As for the inclusion criteria, patients had to have a diagnosis of MS according to the revised McDonald criteria (Thompson et al., 2018) and a relapsing-remitting phenotype; moreover, they had to be relapse- and corticosteroid-free within the month prior to scanning. As for the HCs, no T2 hyperintense lesions had to be shown in the MRI scanning. Both MS patients and HCs had to be between 18 and 65 years old, with no history of psychiatric illness and without deficit of oral comprehension defined by an age- and education-adjusted score on the Token Test (Spinnler and Tognoni, 1987) < 26.25.

All participants signed an informed consent form, and the study was approved by the local Ethical Committee.

### Clinical, Neurological, and Behavioral Assessment

All participants underwent a neurological, neuropsychological, and behavioral assessment. All patients underwent Expanded Disability Status Scale (Kurtzke, 1983) to assess the severity of physical disability in MS patients. Patients and HC underwent Symbol Digit Modalities Test (SDMT; Rao et al., 1991; Amato et al., 2006) assessing the information processing speed. SDMT has been recognized as the most sensitive test for screening cognitive impairment in MS (Van Schependom et al., 2014). Moreover, patients and HC completed the Italian version of the Beck Depression Inventory—II Edition (Beck et al., 1996; Sacco et al., 2016) to evaluate depressive symptomatology.

### Social Cognition Abilities

Patients and HCs underwent the ToM Picture Sequencing Task (TMPS; Brüne, 2003) and the Reading the Mind in the Eyes test (RMET; Baron-Cohen et al., 1997; Vellante et al., 2013). The TMPS consists of six sequences of pictures (each sequence includes four cards) depicting stories of cooperation, deception, or cooperation of two characters in deceiving a third person. Cards were shown to participants in a non-logical order, and subjects were asked to order the cards in a logical sequence of events. The sequence ordered by the participant and the seconds taken to complete each task were registered by a trained psychologist. If the story was put in the wrong order by the participant, the psychologist rearranged the figures in the right order before proceeding with a questionnaire that evaluated various aspects of social understanding, such as the person’s levels of belief and false-belief reasoning, which is based on the idea that an individual’s belief may differ from the reality (Ward et al., 2013), and the three different levels of social understanding characterized by an increasing order of complexity: the first-order Theory of Mind (ToM; the ability to discriminate between a person’s and others’ beliefs and mental states), the second-order ToM (the ability to make inferences on other person’s beliefs about the mental states of a third person; Miller, 2009), and the third-order ToM (the ability to infer the mental state of others in complex social interactions; Kumfor et al., 2017). Moreover, the TMPS evaluates the comprehension of basic (i.e., the understanding of the reality) and sophisticated cognitive capacities (i.e., the understanding of deception and the norm of reciprocity) related to social cognition (Mazza et al., 2012). Total score of TMPS ranges from 0 to 59, with higher scores indicating better performance.

The RMET consists of 36 pictures of people’s eyes shown to the participants, surrounded by four words indicating mental states. Participants were asked to choose which word best described the mental state of the person shown in the picture. Total score ranges from 0 to 36, with higher scores indicating better performance.

### MRI Acquisition

Brain MRI scans were acquired on a 3T GE Medical System (Milwaukee, WI, USA) scanner equipped with an eight-channel parallel head coil. The following images were acquired:

(1) Proton density (PD)/T2 weighted [dual-echo fast spin echo, repetition time (TR) = 3.080 ms, echo time (TE)1/TE2 = 24/127.5 ms, slice number = 44 (PD)/44(T2), matrix = 256 × 384, axial slices, field of view (FOV) = 240 mm, slice thickness = 3 mm, interslice gap = 0 mm]; (2) high-resolution 3D-T1 (magnetization-prepared fast spoiled gradient echo, TR = 6.988 ms, TI = 650 ms, TE = 2.85 ms, slice number = 166, matrix = 256 × 256, sagittal slices, flip angle = 8°, FOV = 256 mm, voxel size = 1 × 1 × 1.2 mm^3^); (3) RS-fMRI consisting of 240 volumes of a repeated gradient-echo echo planar imaging T2*-weighted sequence (TR = 1.508 ms, axial slices = 29, matrix = 64 × 64, FOV = 256 mm, slice thickness = 4 mm, interslice gap = 0 mm). During the functional scan, subjects were asked to stay motionless, awake, and relaxed, and to keep their eyes closed.

### Conventional MRI Analysis

The identification of T2 hyperintense lesions in MS patients was conducted on PD/T2 images by a single experienced observer (MC) blinded to the patients’ clinical characteristics. The Medical Image Processing, Analysis, and Visualization (MIPAV) software (version 4.2.2^1^) was used to contour lesions and to compute T2 lesion volume for each patient. Normalized brain (NBV), WM (NWMV), and GM (NGMV) volumes were measured on 3D-T1 images using the SIENAx software, after T1-hypointense lesion refilling (Jenkinson et al., 2012).

### RS-fMRI Analysis (Esposito et al., 2008; Bonavita et al., 2017)

Standard image data preparation and preprocessing, statistical analysis, and visualization were performed with the software BrainVoyager QX (Brain Innovation BV, Maastricht, Netherlands). Data preprocessing included the correction for slice scan timing acquisition, a three-dimensional rigid-body motion correction based on a six-parameter rigid body alignment to correct for minor head movements, and the application of a temporal high-pass filter with cut off set to three cycles per time course. Translational motion parameters were verified to be always less than one functional voxel for all included participants. Structural and functional data were coregistered and spatially normalized to the Talairach standard space using a 12-parameter affine transformation. During this procedure, the functional images were resampled to an isometric 3-mm grid covering the entire Talairach box. Single-subject and group-level independent component analysis (ICA) were carried out on the pre-processed functional time series using two plug-in extensions of BrainVoyager QX, implementing fast ICA algorithm and the self-organizing group-level ICA algorithm, respectively. For each subject, 40 independent components, corresponding to one sixth of the number of time points, were extracted. All single-subject component maps from all subjects were then “clustered” at group level, resulting in 40 single-group average maps that were visually inspected for recognition of the main physiological RS components. The sign-adjusted ICA components of all subjects were then submitted to a second-level, multi-subject random-effects two-way ANOVA that treated the individual subject map values as random observations at each voxel (Esposito et al., 2008), cluster membership as one within-subject factor with 40 levels (corresponding to 40 group components), and subject group as one between-subject factor with two levels (corresponding to HC and MS patients). Starting from ANOVA, a single-group one-sample *t*-test was used to analyze in each group the whole-brain distribution of the main physiological and cognitive RS components: the DMN, left and right FPN (LFPN and RFPN), EN, SLN, LN, and CN components. The resulting *t-maps* were thresholded at *p* = 0.05 (Bonferroni corrected over the entire brain). From these, an inclusive mask was also created from the HC group maps and used to define a new search volume for within-network, two-group comparisons. The voxel-wise comparisons between the two groups were indeed performed with two-sample *t*-test over the search volume. All the comparisons were made with gender and age included as covariate of no interest. To correct the resulting t-maps for multiple comparisons, regional effects within the search volume were considered significant only for compact clusters after the joint application of a voxel- and cluster-level threshold. The cluster-level threshold was estimated non-parametrically with a randomization approach: starting from an initial (uncorrected) threshold of *p* = 0.001 applied to all voxels, a minimum cluster size was calculated that protected against false-positive clusters at 5% after 1,000 Monte Carlo simulations (Forman et al., 1995). Individual ICA z-scores from DMN, LFPN, RFPN, EN, SLN, LN, and CN regions identified in the above analysis were also extracted and used in linear correlation analysis in the MS patients and in the HC groups with several scores: RMET, TMPS total, TMPS first-order ToM, TMPS second-order ToM, and TMPS reciprocity score. For these regional analyses, we used a statistical significance level of *p* < 0.05 (uncorrected). ICA z-scores express the relative modulation of a given voxel by a specific ICA component and hence reflect the amplitude of the correlated fluctuations within the corresponding functional connectivity network.

### Voxel-Based Morphometry Analysis (Good et al., 2001)

Voxel-based morphometry analysis was performed using SPM12 software (Wellcome Trust Centre for Neuroimaging, London, UK^2^) on 3D-T1 lesion-filled images. Images were bias-corrected, tissue-classified, and registered using linear (12-parameter affine) and non-linear transformations (warping) within a unified mode, with default parameters incorporating the DARTEL toolbox (Ashburner, 2008). Subsequently, the warped GM segments were affine-transformed into MNI space and were scaled by the Jacobian determinants of the deformations to account for the local compression and stretching that occurs as a consequence of the warping and affine transformation (modulated GM volumes). The modulated volumes were smoothed with a Gaussian kernel of 8-mm full-width at half maximum. The GM volume maps were statistically analyzed using the general linear model based on Gaussian random field theory. Regional differences in GM volume between the experimental groups (HC vs. MS patients) were assessed with total intracranial volume, age, and sex as covariates of no interest. Correlations between GM volume and RMET, total TMPS, TMPS first-order and second-order ToM, and TMPS reciprocity scores were assessed both in the MS and HC groups using multiple regression analysis with total intracranial volume, age, and sex as covariates of no interest. Statistical inference was performed at the voxel level, with an FWE correction for multiple comparisons. Clusters were considered significant at *p* < 0.05.

A conversion table between Talaraich and MNI space coordinates was added as Supplementary Table S1.

### Statistical Analysis

A Kolmogorov–Smirnov test was used to verify normal distribution of demographic, clinical, and conventional MRI variables. Between-group comparisons were performed using the Mann–Whitney and chi-square tests, as appropriate. Correlations between scores on social cognition tasks and conventional MRI variables were assessed using Spearman’s rank correlation coefficient. A *p* < 0.05 was considered statistically significant; however, a Bonferroni correction for multiple comparisons applied to 10 measures (Beck Depression Inventory—II Edition, SDMT, NBV, NGMV, NWMV, RMET, TMPS, first-order, second-order, and reciprocity scores of TMPS; *p* = 0.005) was performed (SPSS Statistics version 25.0).

## Results

### Clinical/Demographic, Neuropsychological and Conventional MRI Data

**Figure 1:**
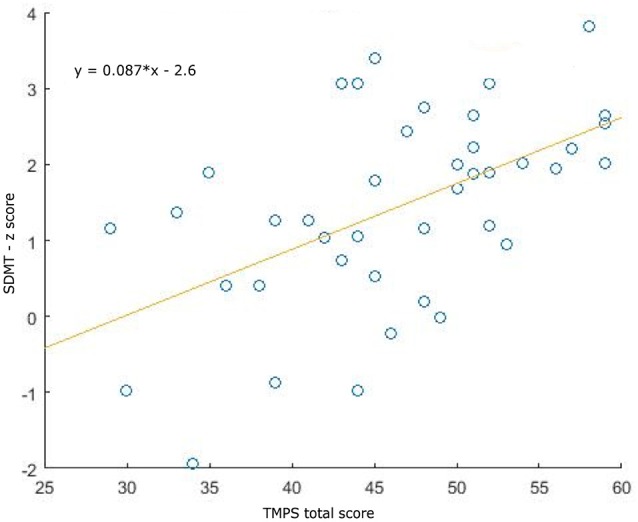
Association plots between Symbol Digit Modalities Test (SDMT) and total Theory of Mind Picture Sequencing Task (TMPS) scores in the multiple sclerosis (MS) patients group.

Clinical, demographical, and MRI characteristics of HC and MS patients groups are described in Table 1. MS patients were cognitively preserved and did not report depressive symptoms. No differences were found between MS patients and HC on cognition and depressive symptomatology; as regards the MRI measures, MS patients had lower NBV, NGMV, and NWMV compared to HC (Table 1). Moreover, MS patients had a lower score than HC on the RMET and TMPS tests, second-order ToM subscale, and reciprocity subscale of TMPS (Table 2). After Bonferroni correction, differences between the two groups on NBV, total score on TMPS, and scores on reciprocity subscale of TMPS remained statistically significant. In the MS group, no association was found between RMET and clinical variables, whereas a strong association was found between TMPS and SDMT scores (rho = 0.520; *p* < 0.001); see Table 2 and Figure 1.

**Table 1:** Socio-demographic and clinical characteristics of multiple sclerosis (MS) patients and healthy controls (HC) and group comparisons.

	MS patients (*n* = 41)	HC (*n* = 25)	*p*
Mean age (years, SD)	34.18 (10.27)	37.83 (11.95)	0.284
Sex (M/W)	14/27	7/18	0.504
Mean disease duration (years, SD)	8.8 (8.2)	–	–
Median EDSS (range)	1.5 (0–6.5)	–	–
Mean SDMT z score (SD)	0.955 (1.43)	1.39 (1.36)	0.194
Median BDI-II (SD)	7 (0–18)	4 (0–14)	0.077
Median T2 LV (mm^3^, range)	1,883 (44–34,934)	–	–
Mean NBV (mm^3^, SD)	1,510 (76)	1,569 (64)	**0.002**
Mean NGMV (mm^3^, SD)	843 (50)	870 (47)	*0.027*
Mean NWMV (mm^3^, SD)	667 (37)	698 (41)	*0.008*
Median RMET score (range)	24 (13–30)	26 (17–29)	*0.021*
Median TMPS total score (range)	47 (29–59)	56 (39–59)	**<0.001**
Median TMPS first-order ToM (range)	5 (2–5)	5 (3–5)	0.233
Median TMPS second-order ToM (range)	4 (2–5)	5 (3–5)	*0.009*
Median TMPS reciprocity score (range)	2 (1–3)	3 (2–3)	**<0.001**

*p, probability value; EDSS, Expanded Disability Status Scores; SDMT, Symbol Digit Modalities Test; BDI-II, Beck Depression Inventory II; T2 LV, T2 lesion volume; NBV, normalized brain volume; NGMV, normalized gray matter volume; NWMV, normalized white matter volume; RMET, Reading the Mind in the Eyes test; TMPS, Theory of Mind Picture Sequencing Task; ToM, Theory of Mind. Significant values (*p* < 0.05) are reported in italic. Significant values after Bonferroni correction for 10 measures (*p* < 0.005) are reported in bold*.

**Table 2:** Associations between social cognition measures and clinical, neuropsychological, and MRI data in the MS sample.

	RMET total score	TMPS total score	TMPS–first-order ToM	TMPS–second-order ToM	TMPS–reciprocity score
Age	−0.024 (0.882)	−0.111 (0.496)	−0.107 (0.513)	0.020 (0.901)	0.150 (0.355)
EDSS	0.015 (0.926)	−*0.364 (0.019)*	−0.035 (0.828)	−*0.383 (0.013)*	−0.006 (0.972)
SDMT	0.257 (0.131)	**0.521 (<0.001)**	0.272 (0.109)	*0.396 (0.017)*	0.038 (0.827)
BDI-II	0.024 (0.891)	−0.183 (0.285)	0.240 (0.159)	−0.270 (0.111)	−0.009 (0.959)
T2 LV	−0.025 (0.880)	−0.222 (0.169)	−0.038 (0.814)	−0.253 (0.116)	0.153 (0.345)
NGMV	−0.052 (0.749)	0.150 (0.356)	0.126 (0.438)	0.137 (0.399)	−0.111 (0.495)
NWMV	−0.221 (0.170)	0.078 (0.632)	*0.328 (0.039)*	0.140 (0.388)	−0.026 (0.873)
NBV	−0.100 (0.539)	0.157 (0.334)	0.238 (0.139)	0.201 (0.215)	−0.083 (0.610)

*RMET, Reading the Mind the Eyes test; TMPS, Theory of Mind Picture Sequencing Task; EDSS, Expanded Disability Status Scores; SDMT, Symbol Digit Modalities Test; BDI-II, Beck Depression Inventory II; T2 LV, T2 lesion volume; NGMV, normalized gray matter volume; NWMV, normalized white matter volume; NBV, normalized brain volume. Values are presented as rho (*p*). Significant values are reported in italic. Significant values after Bonferroni correction for eight measures are reported in bold*.

### RS-fMRI

**Figure 2:**
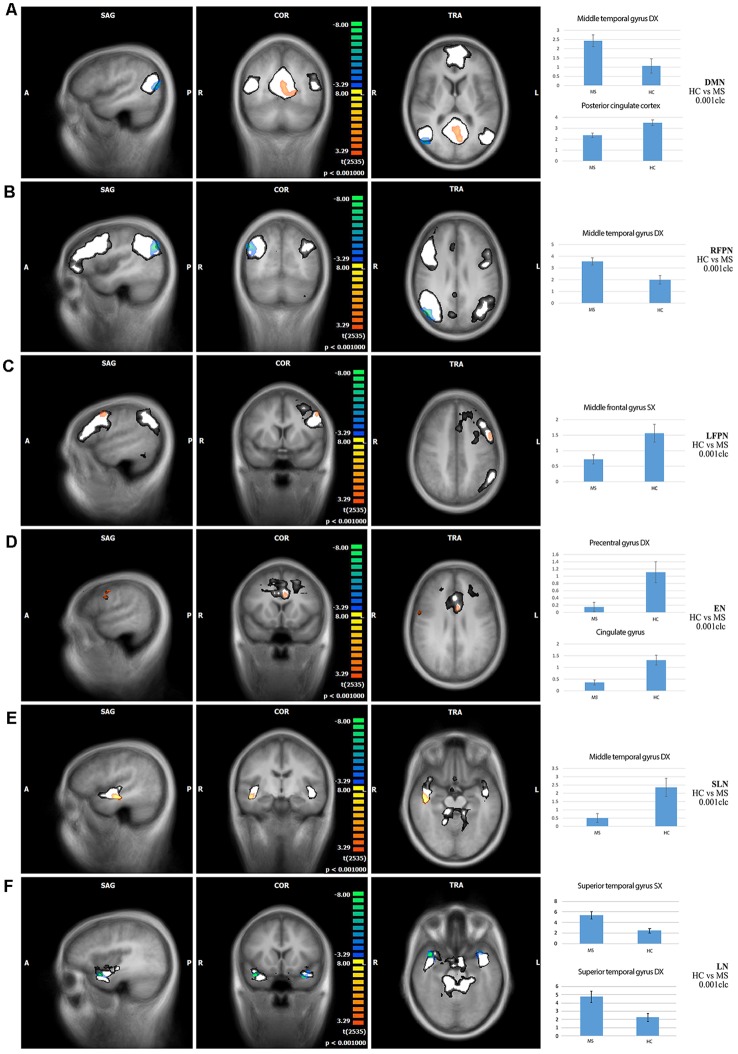
Comparisons of resting-state functional connectivity between healthy controls (HCs) vs. MS patients (first three columns) and bar graphs showing independent component analysis (ICA) scores of highlighted clusters in HC and MS patients (fourth column) in **(A)** default mode network (DMN), **(B)** right fronto-parietal network (RFPN), **(C)** left fronto-parietal network (LFPN), **(D)** executive network (EN), **(E)** salience network (SLN), and **(F)** limbic network (LN). Color legend: hot colors: HC > MS; cool colors: MS > HC; white: explored functional brain network. A: anterior; P: posterior; L: left; R: right; S: superior; I: inferior; SAG: sagittal; TRA: transverse; COR: coronal. The clusters of significant differences (*p* < 0.05, corrected) are overlaid on three orthogonal slices of the averaged normalized anatomy and on the corresponding functional brain networks (in white).

Comparison of RS-FC between MS patients and HC: (1) DMN: MS patients showed a decreased RS-FC in the PCC and an increased RS-FC in the right middle temporal gyrus; (2) RFPN: MS patients showed an increased RS-FC in the right middle temporal gyrus; (3) LFPN: MS patients showed a decreased RS-FC in the left middle temporal gyrus; (4) EN: MS patients showed a decreased RS-FC in the right precentral gyrus and in the cingulate gyrus; (5) SLN: MS patients showed a decreased RS-FC in the right middle temporal gyrus; (6) LN: MS patients showed an increased RS-FC in the right and the left superior temporal gyrus; and (7) CN: no significant differences were found between HC and MS patients (Figure 2).

**Figure 3:**
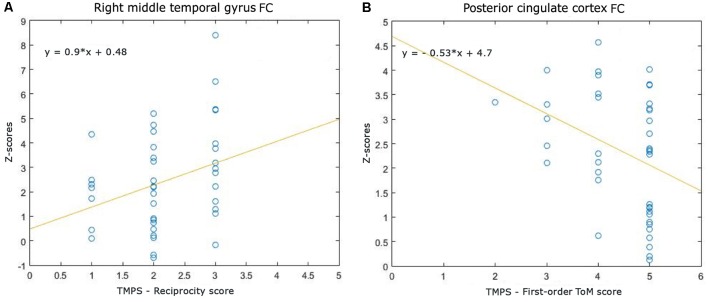
Scatterplots of associations between resting-state functional connectivity (FC) of DMN abnormalities and ToM tasks in MS patients. **(A)** Association between right middle temporal gyrus FC and reciprocity score of TMPS; **(B)** Association between posterior cingulate cortex (PCC) FC and first-order ToM score of TMPS.

**Figure 4:**
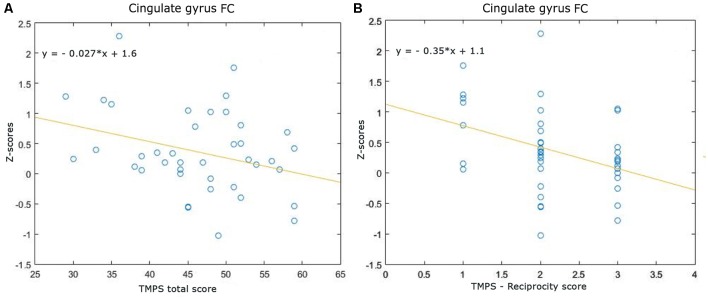
Scatterplots of associations between resting-state functional connectivity (FC) of executive network abnormalities and social cognition tasks in MS patients. **(A)** Association between cingulate gyrus FC and TMPS; **(B)** association between cingulate gyrus FC and reciprocity score of TMPS.

**Figure 5:**
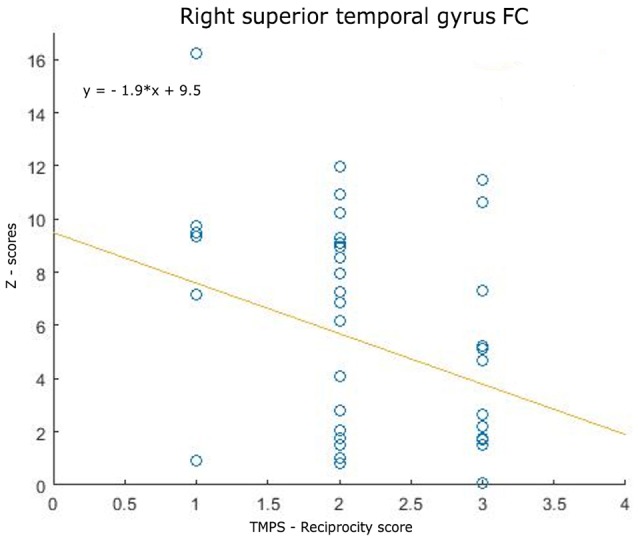
Scatterplot of the association between resting-state functional connectivity (FC) of limbic network abnormalities (right superior temporally gyrus FC) and reciprocity score of TMPS in MS patients.

Correlations between RS-FC networks abnormalities and social cognition tasks in MS patients: (1) DMN (see Figure 3): positive association between RS-FC in the right middle temporal gyrus and score on reciprocity subscale of TMPS (rho = 0.3107, *p* = 0.0480); negative association between RS-FC in the PCC and score on first-order false-belief ToM subscale of TMPS (rho = −0.3318, *p* = 0.0341); (2) EN (see Figure 4): negative association between RS-FC in the cingulate gyrus and total score on TMPS (rho = −0.3161, *p* = 0.0441); negative association between RS-FC in the cingulate gyrus and score on reciprocity subscale of TMPS (rho = −0.3640, *p* = 0.0193); (3) LN (see Figure 5): negative association between RS-FC in the right superior temporal gyrus and reciprocity subscale of TMPS (rho = −0.309, *p* = 0.0493; Figure 4). No correlations were found between social cognition tasks and RFPN, LFPN, and SLN RS-FC abnormalities. In the HC group, reciprocity subscale of TMPS was also negatively correlated with abnormalities in the right middle temporal gyrus in DMN (rho = −0.4668, *p* = 0.0186), while first-order ToM subscale of TMPS was positively correlated with FC in the right precentral gyrus in the EN (rho = 0.3958, *p* = 0.0501).

All detected RS-FC abnormalities did not co-localize with regional GM atrophy.

### Voxel-Based Morphometry Analysis

MS patients, when compared to HC, showed significant regional GM loss in the following areas: the thalamus, bilaterally (right thalamus: *x* = 11, *y* = −30, *z* = −5, *K* = 573; left thalamus: *x* = −8, *y* = −26, *z* = −5, *K* = 1,128), and the superior frontal gyrus (*x* = 35, *y* = 63, *z* = −9, *K* = 208). No areas of regional GM volume reduction were found in HC when compared to MS patients. No correlations between social cognition tasks and regional GM volume were found in MS patients.

## Discussion

In the present cross-sectional study, we aimed to investigate the RSN abnormalities associated with social cognition deficits in MS patients. To our knowledge, this is the first work that used an RS-fMRI approach to explore the relationship between RSN abnormalities and multiple aspects of social cognition. The results indicated a significant association between RS-FC abnormalities in the DMN, EN, and LN and poor performance on TMPS in MS. Only TMPS task was found to be strongly associated with SDMT, a cognitive task measuring processing speed and attention. These results partially replicate previous studies; for example, Raimo et al. (2017) found significant and strong associations with SDMT scores with several aspects of social cognition.

As regards the association between RS-FC and social cognition tasks, the results confirm that DMN is involved in social cognition abilities in MS, consistently with other previous studies with healthy subjects and with patients affected by neurological diseases, such as amyotrophic lateral sclerosis (Li et al., 2014; Trojsi et al., 2017). In more detail, we observed that more difficulties in understanding the concept of the social norm of reciprocity in interpersonal relationships were associated with reduced RS-FC in the right middle temporal gyrus, which belongs to DMN. Our results are in line with a recent meta-analysis including task-based fMRI studies on healthy subjects who showed an activation of the right middle temporal gyrus during the execution of social cognition tasks that assessed false beliefs and rational actions (Schurz et al., 2014). Moreover, the same association was found in our HC group, although with an opposite direction of correlation (a negative association in the HC group vs. a positive association in the MS group). This may suggest the presence of an attempt to compensate social cognition deficit in MS patients or a maladaptive mechanism: the possible disruption of parts of neural circuits could increase the FC of other brain areas that, in absence of lesions, show lower FC.

In the present study, we also observed a negative association between another node of the DMN, the PCC, and performance on first-order ToM: patients with an increased connectivity in this node performed worse on tasks that require differentiating between their own and others’ mental states. PCC is involved in outcome monitoring and in social cognition as revealed in studies performed on HC and in patients with neurological diseases, such as amyotrophic lateral sclerosis (Maddock et al., 2003; Kable and Glimcher, 2007; Trojsi et al., 2017). Moreover, the involvement of PCC in social cognition in MS was also confirmed by a previous fMRI meta-analytic study where an activation of the PCC was described during the execution of non-verbal false belief stories tasks (Schurz et al., 2014). The negative association found in MS patients between PCC and first-order social cognition might imply a maladaptive attempt to compensate social cognition deficits in MS patients or an unbalancing in DMN network as counterpart of the middle temporal gyrus hyperconnectivity.

Another interesting finding of the present study was the association between a lower performance on TMPS, in particular on the reciprocity subscale, and an increased connectivity in the cingulate gyrus of the EN. The results might suggest a maladaptive role of EN abnormalities on social cognition in MS. Although in the literature there are no studies that associate the EN with social cognition, the cingulate gyrus—in particular the ACC—has been related to error detection, monitoring of conflict, cognitive control (Carter et al., 1998; Posner and DiGirolamo, 1998; Bush et al., 2000), and also cognitive flexibility, which has a relevant role in taking other people’s perspective (Leber et al., 2008; Champagne-Lavau et al., 2012). Indeed, in our sample of MS patients, the increased connectivity of the cingulate gyrus may lead to difficulties in attribute mental states such as goals or intentions to others. These difficulties may be due to the damage of part of the brain network secondary to the diffuse presence of MS WM lesions.

Lastly, we observed an association between the right superior temporal gyrus in the LN and the reciprocity score of TMPS. The role of the right superior temporal gyrus in social cognition was confirmed in other studies; for example, Schurz et al. (2014) reported a consistent connectivity of the right superior temporal gyrus in several social cognition tasks, mainly related to false-belief reasoning, trait judgment, and social interactions. Moreover, abnormalities in the right superior temporal gyrus were found in a sample of children and adolescents with autism spectrum disorder, a neurodevelopment disorder characterized by impaired social cognition abilities (Jou et al., 2010). In MS patients, however, we found a negative association between the increased connectivity of the right superior temporal gyrus in the LN and the performance on reciprocity subtest of TMPS. As stated above, this may be due to the occurrence of compensatory processes to suppress social cognition deficits caused by GM and WM lesions or maladaptive mechanisms (Chalah and Ayache, 2017).

It should be noted that all the significant associations between abnormalities in RS-FC and social cognition tasks were mostly moderate, highlighting the complexity of the phenomenon we have explored. Since we have shown only the contribution of FC at rest, we cannot provide a final explanation for these results. We may argue that the social cognition deficits are probably due to the coexistence of structural (Mike et al., 2013; Batista et al., 2017a,b; Chalah et al., 2017) and functional abnormalities. Moreover, other variables may moderate the relationship between abnormalities in RS-FC and ToM tasks, such as cognitive reserve, which is considered a protective factor against the impact of brain damage on specific cognitive abilities in MS patients (Santangelo et al., 2019). The study of such variables might prove an important area for future research.

With regard to the various components of social cognition assessed in this research, only the TMPS, that evaluates belief and false-belief reasoning and the comprehension of basic and sophisticated cognitive capacities, was related to RS-FC abnormalities of DMN and EN, while we did not find any significant associations between the RMET, evaluating the ability to understand others’ emotional states, and RS-FC abnormalities of brain networks; differences in methodology (RS vs. task-based fMRI) and the low sample size of previous studies might explain these discrepancies (Krause et al., 2009; Jehna et al., 2011).

In our study, we did not find any significant association between global or focal GM atrophy and social cognition tasks, while previous studies did, evidencing a relationship between social cognition and GM atrophy in bilateral regions of the orbitofrontal cortex, insula, fusiform gyrus, praecuneus, cingulate cortex, and amygdala (Batista et al., 2017a; Chalah et al., 2017; Ciampi et al., 2018). We were not able to replicate the results of previous studies; this inconsistency may be explained by differences in the sample of MS patients, which differed for clinical variables, such as the phenotype or the cognitive status of the patients.

Social cognition is a broad construct that includes different cognitive processes related to social interaction (Beer and Ochsner, 2006). Several theoretical models have been proposed to describe the processes involved in social cognition and how these processes relate to each other; for example, according to Arioli et al. (2018), social cognition includes three main domains: social perception (the ability to distinguish between objects and persons), social understanding (the ability to decode others’ behaviours) and social decision making (the ability to make decisions on the basis of others’ behaviours). As for the relationships between processes, there are models that distinguish between cognitive ToM, affective ToM and empathy (Shamay-Tsoory et al., 2010), but there are also evidences of overlapping constructs and topographical convergences across brain activities related to these processes (Bzdock et al., 2012). These theoretical assumptions or evidences also reflect the difficulties in evaluating and differentiate between each single component of social cognition: for example, the RMET has been considered as a measure of ToM (Baron-Cohen et al., 1997; Vellante et al., 2013) or cognitive empathy (Warrier et al., 2017) by different authors.

Although the instruments that we employed in our study can be roughly included in the domain of social understanding, given the methodological difficulty to assess the single components of social cognition, it must be taken into account that some processes here assessed (i.e., cognitive empathy vs. ToM) may overlap. Therefore, future studies could further expand our results by employing questionnaires and tools that measure multiple domains of social cognition and that are able to better discriminate between these processes.

This study is not exempt from limitations. First, we were not able to recruit an equal number of patients and controls (41 vs. 25, respectively). Moreover, when testing group differences between MS patients and HC on social cognition tasks, only total score on TMPS and score on the reciprocity subscale of TMPS remained significant after the Bonferroni correction. Nevertheless, we decided anyway to test the correlations of all social cognition tasks with RSN abnormalities because of the explorative nature of our study. In addition, since a too strict Bonferroni correction for multiple comparisons would have negatively influenced the explorative nature of the study, unadjusted *p*-values were considered. Moreover, while we employed two tests that evaluated social cognition in non-verbal modality, we could not assess the relationship between the RS-FC of cognitive-related brain networks and social cognition tasks in verbal modality. This issue should be investigated in future studies. Finally, our sample included MS patients with a medium disease duration, with no cognitive impairment, and with a relatively low lesion load; because of the nature of our cross-sectional study, we could not evaluate the evolution of the associations with time, in later stages of the disease. Future research should be conducted by taking into account the limitations and the methodological weakness of the present study; we believe that the use of a larger sample, employing statistical corrective measures to rule out the possible presence of a type 1 error, and using multiple psychological tests that are able to assess several components of social cognition and differentiate between the several processes that are part of social cognition might confirm and expand our novel findings.

In conclusion, the present study reinforces and expands the notion that patients with MS exhibit an impairment of social cognition abilities. Moreover, the performance on social cognition tasks (in particular those that assess social cognition) appears to be related to specific abnormalities in RSNs related to cognition, such as the DMN, the EN, and the LN. It must be emphasized, however, that the findings of our explorative study have to be treated with caution due to some methodological weaknesses (i.e., some data were not corrected for multiple comparison, different numbers of participants in the two groups). Future investigations should confirm our results by employing a larger number of patients, while also adopting a longitudinal design.

Among the subscales of TMPS, the reciprocity score was significantly associated to three nodes in three different brain networks (DMN, EN, and LN). The social norm of reciprocity plays a key role into maintaining cooperation among members of society; it is now considered a common feature of social cognition, since recent literature confirmed that social cognition is not only a process linked to “pure observation” of social situations, but it also includes elements of social interaction (Bratman, 2013). This component of social cognition seems to be particularly impaired in MS patients, as demonstrated in our study. Since social cognition abilities are necessary to maintain good social relationship, and MS patients need a good social support in order to reduce the risk of developing depression or anxiety (Schwartz and Frohner, 2005; Feinstein et al., 2013), our findings can have clinical implications. Indeed, a better understanding of the mechanism subtending social cognition impairment in MS patients might prompt clinicians to identify patients at risk and provide them with specific and personalized psychoeducational programs to enhance or maintain their social cognition abilities and thus to help them to preserve their social relationships.

## Data Availability Statement

The datasets generated for this study are available on request to the corresponding author.

## Ethics Statement

The studies involving human participants were reviewed and approved by Comitato Etico Azienda Ospedaliera Universitaria Luigi Vanvitelli—AORN Ospedale dei Colli. The patients/participants provided their written informed consent to participate in this study.

## Author Contributions

AG, GS, and AB conceived and designed the study. MA, SP, RC, and Ad’A collected clinical/neuropsychological data. MC collected MRI data. MA, FD, GC, and RD performed statistical and MRI analysis. AB and MA wrote the manuscript. AG, GS, and GT supervised the project. FE, SB, and FT contributed to the interpretation of the results and to the final version of the manuscript (provided critical feedback and revised the manuscript). All authors discussed the results and contributed to the final manuscript.

## Conflict of Interest

AB has received speaker honoraria and/or compensation for consulting service from Biogen, Merck, and Genzyme. MA, GS, FD, RD, GC, RC, SP, Ad’A, MC, FE, and FT have no disclosures. SB has received speaker honoraria and advisory board fees from Novartis, Roche, Merck Serono, Biogen, Teva, Genzyme, and Almirall. GT has received compensation for consulting services and/or speaking activities from Biogen, Novartis, Merck, Genzyme, Roche, and Teva, and receives research support from Biogen Idec, Merck Serono, and Fondazione Italiana Sclerosi Multipla. AG received honoraria for speaking and travel grants from Biogen, Merck, Genzyme, Teva, Mylan, Roche, and Novartis.

## Footnotes

^1^http://mipav.cit.nih.gov

^2^http://www.fil.ion.ucl.ac.uk/spm
