# Supplementary material for: Resting-State Functional Correlates of Social Cognition in Multiple Sclerosis: An Explorative Study
Source: Front Behav Neurosci. 2020 Feb 6;13:276. doi: 10.3389/fnbeh.2019.00276 (PMC7016209; doi:10.3389/fnbeh.2019.00276)
Supplement: Supplementary file 1 [file Table_1.pdf]

Supplementary Material

Supplementary material 1. Conversion table between Talairach and MNI space coordinates

**ROI**	**TAL**	**MNI**
DMN – Right middle temporal gyrus	44 -71 12	43 -73 9
DMN - PCC	-4 -53 6	-5 -54 2
RFPN – Right middle temporal gyrus	44 -68 30	43 -71 31
LFPN - Left middle frontal gyrus	-49 7 39	43 -71 31
EN – Right precentral gyrus	57 4 33	-50 6 42
EN – Cingulate gyrus	-34 1 49	-34 -1 53
SLN – Right superior temporal gyrus	44 -14 -12	47 -12 -19
LN – Right superior temporal gyrus	38 10 -18	40 16 -26
LN – Left superior temporal gyrus	-31 13 -22	-31 19 -28

ROI = Region of interest; TAL = Talairach coordinates; MNI = Montreal Neurological Institute coordinate system
